# Rate of Changes in CMT Neuropathy and Examination Scores in Japanese Adult CMT1A Patients

**DOI:** 10.3389/fneur.2020.00626

**Published:** 2020-07-16

**Authors:** Fukiko Kitani-Morii, Yu-ichi Noto, Yukiko Tsuji, Kensuke Shiga, Ikuko Mizuta, Masanori Nakagawa, Toshiki Mizuno

**Affiliations:** ^1^Department of Neurology, Graduate School of Kyoto Prefectural University of Medicine, Kyoto, Japan; ^2^Department of Neurology, Matsushita Memorial Hospital, Osaka, Japan; ^3^Department of Neurology, North Medical Center, Kyoto Prefectural University of Medicine, Kyoto, Japan

**Keywords:** Charcot-Marie-Tooth disease (CMT1A), CMTNS, CMTES, older, progression

## Abstract

**Introduction:** We aimed to clarify when adult patients with Charcot-Marie-Tooth disease type 1A (CMT1A), especially those diagnosed at middle or advanced ages, first showed symptoms and whether the rate of disease progression is accelerated by aging.

**Methods:** Medical records of CMT1A outpatients between 2012 and 2019 were reviewed. The age at diagnosis, age when symptoms first appeared, and rate of disease progression, assessed based on clinical outcome measures including the CMT Neuropathy Score (CMTNS), Rasch-modified CMTNS (CMTNS-R), CMT Examination Score (CMTES), and Rasch-modified CMTES (CMTES-R) were analyzed.

**Results:** Among 45 adult CMT1A patients, 42% had been diagnosed after 50 years of age, whereas 91% of all patients had exhibited some CMT-related symptoms before 20 years of age. The annual increase of all clinical outcome measures did not differ between patients under and over 50 years. Even when limited to patients whose initial CMTES-R showed mild to moderate severity, the rate of change in CMTES-R did not differ between the two age groups (the annual mean ± standard deviation, under 50 years: 1.1 ± 1.0, and over 50 years: 0.9 ± 1.1, *p* = 0.68). To determine whether patients with disabilities at a young age have a higher deterioration rate, they were classified into three groups according to their current age and age at diagnosis: patients under 50 years of age, patients over 50 years of age but diagnosed before 50, and patients diagnosed after 50 years of age. The mean annual increase of all clinical outcome measures, however, did not differ among these groups (CMTES-R: 1.03 ± 1.01 vs. 0.94 ± 1.57 vs. 0.81 ± 0.88, respectively, *p* = 0.87).

**Discussion:** CMT1A patients develop symptoms in childhood and adolescence even if such symptoms are not noticeable until reaching an advanced age. Deterioration rates of clinical outcome measures are constant irrespective of the age in their adulthood, although we cannot rule out the limitation that the difference did not reach significance because of the small number of patients. Being aware of the existence of a considerable number of undiagnosed CMT patients will help promote the avoidance of inadequate medication.

## Introduction

Charcot-Marie-Tooth (CMT) disease is one of the most common inherited peripheral neuropathies, with more than 80 known causative genes (1, 2). Among various CMT subtypes, CMT1A is the most prevalent genetic form, which constitutes about 60% of patients with genetic diagnosis (3). CMT1A is caused by the duplication of chromosome 17p11.2, which contains the *Peripheral Myelin Protein 22-kDa* (*PMP22*) gene (4). Predominant symptoms are slowly progressive distal muscle atrophy, sensory loss, and foot deformities. The disease onset is usually in the first or second decade of life (5); however, not all patients are diagnosed in childhood. Indeed, there have been several reports of undiagnosed adult CMT patients found to have the disease after the exacerbation of neurological symptoms due to medications (6–8). It was reported that such patients had, in retrospect, shown overt CMT-related features before receiving medications. Undiagnosed adult CMT patients are considered to be young at onset; however, there is no precise information on whether patients diagnosed with CMT in adulthood, especially at middle or advanced ages, develop the disease in the first or second decade of life.

Whether the disease progression rate increases with aging is also a matter of debate. Some authors reported that the rate of disease progression is relatively constant, while others indicate that deterioration is accelerated by aging (9–12). If aging accelerates progression, we should be more careful in the clinical management of older CMT1A patients.

We conducted this retrospective study to investigate when adult CMT1A patients, especially those diagnosed at middle or advanced ages, first showed symptoms and whether the rate of disease progression is affected by aging.

## Materials and Methods

We retrospectively analyzed the clinical course of adult CMT1A patients by reviewing medical records. The study protocol was reviewed and approved by the institutional ethics review boards of Kyoto Prefectural University of Medicine.

### Patients

We collected clinical data on all consecutive CMT1A patients aged 20 years or older who visited the Neurology Clinic of Kyoto Prefectural University of Medicine between 2012 and 2019. The diagnosis of CMT1A was based on the results of family history-taking and genetic testing for *PMP22* duplication using fluorescence *in situ* hybridization (FISH, LSI Medience Corporation, Tokyo, Japan). Medical interview, physical examination, and a nerve conduction study had been conducted at least once for all patients. Trained neurologists performed history-taking and physical examination. Board-certified clinical neurophysiologists carried out nerve conduction studies.

### Clinical Parameters

Demographic data including age and sex, past medical history and family history were reviewed. In this study, we collected information about “the age at diagnosis,” which was defined as when a patient diagnosed with CMT1A. Additionally, to investigate when CMT-related symptoms appeared, we collected information about “the age at which symptoms first appeared,” which was defined as the time when patients initially noticed or showed distal dominant sensory-motor impairment. Notably, most Japanese elementary schools conduct annual athletic performance measurements of their students (children aged 6–12 years), and sprinting abilities are also evaluated in most students. For this reason, we asked patients whether they had sprinting difficulties in childhood in addition to classical CMT-related symptoms like foot deformities or gait difficulties.

### Clinical Outcome Measures for CMT

Clinical outcome measures for CMT including the CMT Neuropathy Score (CMTNS, version 2), Rasch-modified CMTNS (CMTNS-R), CMT Examination Score (CMTES), and Rasch-modified CMTES (CMTES-R) were evaluated by trained investigators (13–15). The most recent data were used for analysis of the current status. The disease severity based on CMTES-R was classified into three groups: mild 0–9, moderate 10–18, and severe = or >19 (15). Considering that a previous report suggested that motor decline in CMT1A patients accelerated after 50 years of age (12), patients were divided into three groups according to their current age and age at diagnosis of CMT: (1) patients under 50 years of age, (2) patients over 50 years of age but diagnosed before 50, and (3) patients diagnosed after 50 years of age. The annual changes in CMTNS, CMTNS-R, CMTES, and CMTES-R were calculated as follows: the difference between the initial and most recent data was divided by the observational period (from the date of the initial score to that of the most recent score) to show the change in these scores per year. For analysis of the annual change, we excluded the following patients: those who had received only a single assessment, and those with medical histories potentially influencing neuropathic symptoms (i.e., diabetes mellitus, chemotherapy, and orthopedic surgery) from 2012 to 2019.

### Statistical Analysis

All data are shown as the mean ± standard deviation (SD). The average and annual change of CMTNS, CMTNS-R, CMTES, and CMTES-R during adulthood were analyzed using the Mann-Whitney test for two groups or one-way ANOVA for three groups. Statistical analysis was performed using R version 3.4.2 (www.r-project.org). An alpha value of <0.05 indicated significance.

## Results

### Patients

**Figure 1:**
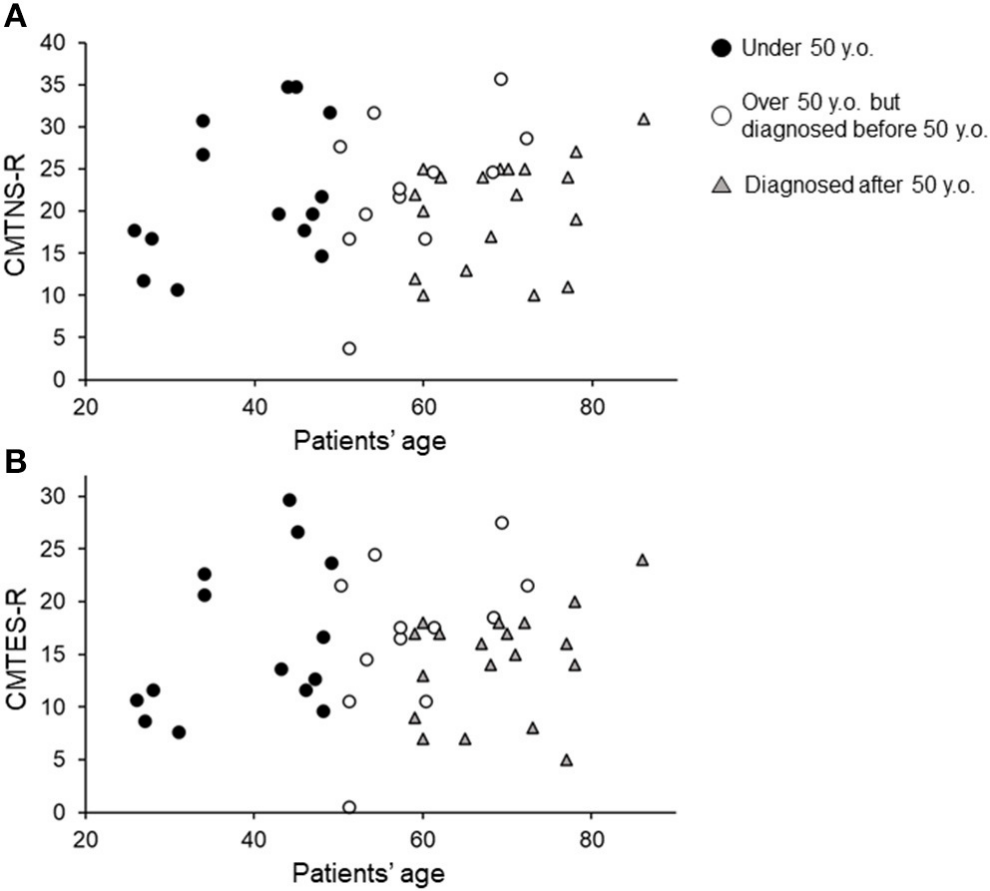
Current CMTNS-R **(A)** and CMTES-R **(B)** distribution classified by current age and age diagnosed with CMT1A. Black filled circles indicate patients under 50 years of age. White circles indicate patients over 50 years of age but diagnosed before 50. Gray triangles indicate patients diagnosed after 50 years of age.

Forty-five patients were identified, and their characteristics are shown in Table 1. Of all patients, 4 had diabetes mellitus, which exacerbates CMT symptoms (16). No patients received chemotherapy or orthopedic surgery from 2012 to 2019. Most patients were ambulatory, and one patient with diabetes mellitus was wheelchair-dependent. Among the 45 patients, 40 directly showed *PMP22* duplication by FISH, and the remaining 5 had parents or siblings with *PMP22* duplication. The distribution of CMTNS-R and CMTES-R were shown in Figure 1. When the patients were classified into three subgroups based on CMTES-R (15), eight patients (17%) were classified as mild (CMTES-R, 0–9), 26 patients (57%) as moderate (10–18), and 11 patients (24%) as severe (> or = 19). Regarding the family history, 10 patients (22%) had relatives already diagnosed with CMT by genetic testing and visited the hospital based on their recommendation, and 9 patients (20%) had relatives who were secondarily suspected of having CMT. Nineteen patients (42%) had no relatives with obvious CMT symptoms, and seven patients (15%) were classified as unknown, because it could not be confirmed whether their family members were affected because of adoption, parents' divorce, or death.

**Table 1:** Patients characteristics.

**Variable**	
Sample size, *n*	45
Sex, % male	42
Age, mean ± SD, y	56.9 ± 15.0
CMTNS, mean ± SD	17.1 ± 6.4
CMTNS-R, mean ± SD	21.7 ± 7.4
CMTES, mean ± SD	11.9 ± 5.5
CMTES-R, mean ± SD	15.5 ± 6.3
**Family history**	
Patients with relatives already diagnosed with CMT, *n* (%)	10 (22)
Patients with relatives secondarily suspected of CMT, *n* (%)	9 (20)
Patients w/o relatives showing CMT symptoms, *n* (%)	19 (42)
Unknown, *n* (%)	7 (15)

*CMT, Charcot-Marie-Tooth; CMTNS, CMT Neuropathy Score; CMTNS-R, Rasch-modified CMT Neuropathy Score; CMTES, CMT Examination Score; CMTES-R, Rasch-modified CMT Examination Score; w/o, without; SD, standard deviation*.

### Age at Which Symptoms First Appeared and Age at Diagnosis

**Figure 2:**
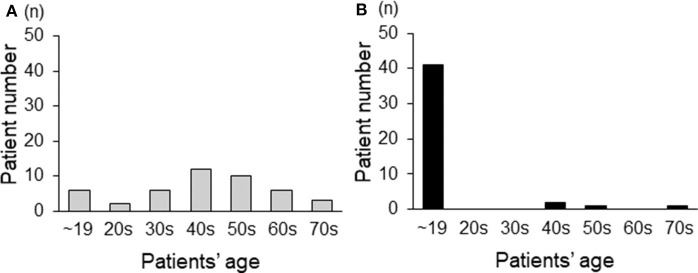
The age distribution of adult CMT1A patients based on the age at diagnosis **(A)** and the age at which symptoms first appear **(B)**.

Regarding CMT-related symptoms in childhood and adolescence, 39 patients (86%) reported that they sprinted much slower than other students in elementary school, 10 patients (22%) were aware of foot deformity in childhood, and 9 patients (20%) reported walking difficulties before 20 years of age. When examining data on the age at diagnosis, 42% (19 patients) of our cohort had been diagnosed after 50 years of age (Figure 2A). On the other hand, regarding the age at which symptoms first appeared, 91% (41 patients) showed some symptoms before 20 years of age (Figure 2B).

### The Annual Change in Clinical Outcome Measures for CMT

A previous report suggested that CMT1A may progress faster after the age of 50 (12); thus we examined whether the annual rate of change in clinical outcome measures (CMTNS, CMTNS-R, CMTES, and CMTES-R) worsened rapidly after the age of 50. Of the total of 45 patients, four patients were excluded because of diabetes mellitus and eight were excluded because of having only single time-point examination data; 33 had multiple data obtained at different time-points. Patients with a single visit showed significantly lower CMTNS-R and CMTES-R than those with multiple visits (CMTNS-R: 15.5 ± 6.9 vs. 23.0 ± 6.9, *p* < 0.01, CMTES-R: 10.2 ± 5.8 vs. 16.6 ± 5.8, *p* < 0.01, respectively), although the mean age was not significantly different between two groups (patients with a single visit: 58.5 ± 9.3 years of age, patients with multiple visits: 56.6 ± 16.0 years of age, *p* = 0.62).

When patients were simply categorized as over 50 years of age and 50 or younger (<50 years of age: younger group, ≥50 years of age: older group), the annual changes of clinical outcome measures were not significantly different between the two groups: younger vs. older group, CMTNS: 0.86 ± 0.82 vs. 0.94 ± 0.96, *p* = 0.40, CMTNS-R: 1.15 ± 1.04 vs. 0.94 ± 1.12, *p* = 0.70, CMTES: 0.75 ± 0.85 vs. 0.80 ± 0.97, *p* = 0.44, and CMTES-R: 1.03 ± 1.01 vs. 0.85 ± 1.12, *p* = 0.67, respectively (Supplementary Table 1). The mean observational period did not differ between the two groups (younger group: 4.7 ± 1.9 years, and older group: 5.2 ± 1.9 years, *p* = 0.74). Because Fridman et al. showed that CMTES-R was sensitive to change in patients diagnosed as mild to moderate but not severe (15), we examined the rate of change between the two groups after excluding patients whose initial CMTES-R was over 19. As a result, a total of 31 patients were evaluated (11 patients were in the younger group, and 20 patients were in the older group), and there was no significant difference in the annual change of CMTES-R between the two groups (younger group: 1.1 ± 1.0, and older group: 0.9 ± 1.1, *p* = 0.68).

**Figure 3:**
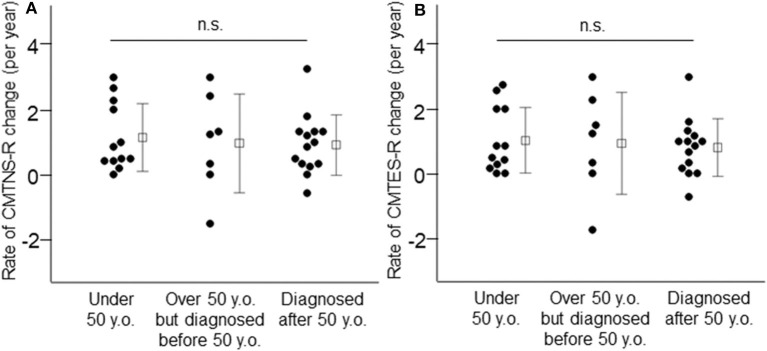
Annual changes of CMTNS-R **(A)** and CMTES-R **(B)** were demonstrated in the three patient groups: patients under 50 years of age, patients over 50 years of age but diagnosed before 50, and patients diagnosed after 50 years of age. White squares indicate mean values and bars indicate standard deviation. n.s.: not significant.

Next, we classified patients into three groups according to their current age and age at CMT diagnosis, to investigate whether patients with more severe symptoms at an early age show a greater rate of progression with aging than those diagnosed with CMT at an advanced age. As shown in Figure 3 and Supplementary Table 1, the average annual change in clinical outcome measures did not differ among these three patient groups (patients under 50 years of age vs. patients over 50 years of age but diagnosed before 50 vs. patients diagnosed after 50 years of age, CMTNS: 0.86 ± 0.82 vs. 1.01 ± 1.17 vs. 0.90 ± 0.89, *p* = 0.94, CMTNS-R: 1.15 ± 1.04 vs. 0.97 ± 1.52 vs. 0.90 ± 0.89, *p* = 0.86, CMTES: 0.75 ± 0.85 vs. 0.91 ± 1.22 vs. 0.75 ± 0.87, *p* = 0.92, CMTES-R: 1.03 ± 1.01 vs. 0.94 ± 1.57 vs. 0.81 ± 0.88, *p* = 0.87, respectively). The mean observational period also did not differ among the three groups (patients under 50 years of age: 5.0 ± 1.9 years, patients over 50 years of age but diagnosed before 50: 4.1 ± 2.4 years, patients diagnosed after 50 years of age: 5.2 ± 1.7 years, *p* = 0.48).

## Discussion

In this study, we identified the following features in our Japanese adult CMT1A cohort. Firstly, approximately 42 percent of patients had been diagnosed with CMT1A after 50 years of age, whereas about 91 percent of all patients had already shown CMT-related symptoms during their first two decades of life. Secondly, the annual change in disease progression during adulthood was constant at least based on CMTNS, CMTNS-R, CMTES, and CMTES-R, irrespective of age.

Some reports already showed that CMT patients could be diagnosed at any age even if they develop the disease at a young age, because patients with mild symptoms were sometimes unaware of having been affected during their childhood (6, 17, 18). Actually, Wojciechowski et al. reported that CMT children with no difficulty on heel or toe walking showed a near-normal gait pattern (19). Among various symptoms of CMT, difficulty running was reported to be present in almost all patients and was one of the symptoms having a major impact on life (20). Although there are no reports detailing the running posture of CMT patients, difficulty running can be caused by foot deformities and muscle weakness either alone or in combination, even if they are not noticeable. Furthermore, Garcia et al. reported CMT1A patients who developed running difficulty as the initial symptom (21). Overall, as noted in this study, a sub-normal sprinting ability is consistent with a mild symptom of CMT1A. Indeed, we revealed that more than 80% of our patients said that they had sprinted much slower than other students in elementary school, as such schools in Japan regularly evaluate children's motor performance, even if they had no difficulties in daily life. Combining with classical symptoms like foot deformities and walking difficulties, more than 90% of our patients had shown CMT-related symptoms before reaching 20 years of age, as shown in Figure 2. These results indicate that almost all CMT patients develop the disease in childhood or adolescence, but such symptoms may be so mild in some patients that they go unnoticed until reaching an advanced age.

In our cohort, the exacerbation rate of clinical outcome measures (CMTNS, CMTNS-R, CMTES, and CMTES-R) in adulthood was constant irrespective of age (Figure 3), even when limited to mild- to moderate-severity patients on calculating CMTES-R (15). According to previous reports, Dyck et al. showed that the annual deterioration rate did not differ between patients aged 14–39 and those aged 40 or more years old in hereditary motor and sensory neuropathy 1a (Neuropathic Deficit Score points, 1.1 and 0.9, respectively) (9). In contrast, Shy et al. suggested a tendency whereby the older the patient, the greater the annual rate of progression based on CMTNS, although it did not reach significance (10). Tozza et al., based on a study with a cross-sectional design, also reported that the deterioration of CMTNS and other functional measures showed an increase in the rate of change after 50 years of age (12). Our results contradict these two reports, but differences in study design may partially explain the discrepancy. In addition, compared with the study of Tozza et al., our younger patients tended to show a higher CMTNS (Figure 1). This may be the reason why our study did not reveal similar changes. On the other hand, Verhamme et al. reported two contradictory results: declines in axonal function and muscle strength were similar in CMT patients and controls, whereas physical disability showed a greater increase over time in patients than controls (11). They stated that skeletal deformations due to muscle weakness may decrease reserves and compensatory mechanisms, and that this may lead to more marked physical disability in adult CMT1A patients. Overall, in adult CMT1A patients, primary pathological changes in peripheral nerves and muscles may be constant, whereas physical performance, requiring the orchestrated interaction of multiple muscles and sensations, deteriorated more markedly over time.

There are some limitations of this study. Firstly, we cannot rule out the possibility that the limited number of patients influenced the results. In other words, because of the small number of patients obtained by subgroup classification by current age and age at diagnosis, the difference in the annual changes of clinical outcome measures with age may not reach a significant difference. Secondly, this was a single-center study, which is associated with a bias in patients' characteristics. Thirdly, although we showed that the annual increase of clinical outcome measures (CMTNS, CMTNS-R, CMTES, and CMTES-R) was constant regardless of age, it will be necessary to validate using other evaluation methods, such as functional and patient-reported outcome measures, as recommended by Rossor et al. (22) To overcome these limitations and answer the question of whether the deterioration rate increases over time, a multi-center study with multiple outcome measures is needed. Finally, because single-visit patients showed significantly lower CMTNS than multiple-visit patients, our cohort may be biased toward severe patients and the rate of annual change may be underestimated by missing such mild cases. However, since there was no significant difference in the average age between the single-visit and multiple-visit groups, it is considered that the effects of missing such mild cases were relatively equal in the two groups.

In conclusion, our study indicates that CMT1A patients in adulthood show no obvious age-related increase in CMTNS, CMTNS-R, CMTES, of CMTES-R. Furthermore, because almost all CMT1A patients develop symptoms in childhood and adolescence, even if they are unaware of them, it is important to be conscious of the existence of a considerable number of undiagnosed adult patients. Early diagnosis may help to ensure the appropriateness of care including physical therapy (23) and avoid CMT-specific adverse events resulting from medications for other diseases (6–8, 24–26).

## Data Availability Statement

The datasets generated for this study will not be made publicly available. The current protocol approved by the local ethics committee of Kyoto Prefectural University of Medicine Current does not permit joint research with other facilities. Requests to access the dataset can be directed to the corresponding author.

## Ethics Statement

The authors confirm that they have read the journal's position on issues involved in ethical publication and affirm that this report is consistent with those guidelines. The patient was evaluated at Kyoto Prefectural University of Medicine hospital under a protocol approved by the local ethics committee of Kyoto Prefectural University of Medicine. Written informed consent was obtained from the patient.

## Author Contributions

FK-M, YN, MN, and TM contributed conception and designs of the study. FK-M organized the database and performed the statistical analysis. YN and YT performed neurophysiological studies. KS, IM, MN, and TM contributed to outpatient care. All authors contribute to manuscript revision, read, and approved the submitted version.

## Conflict of Interest

The authors declare that the research was conducted in the absence of any commercial or financial relationships that could be construed as a potential conflict of interest.
