# Supplementary figures and images for: Rate of Changes in CMT Neuropathy and Examination Scores in Japanese Adult CMT1A Patients
Source: Front Neurol. 2020 Jul 16;11:626. doi: 10.3389/fneur.2020.00626 (PMC7378731; doi:10.3389/fneur.2020.00626)

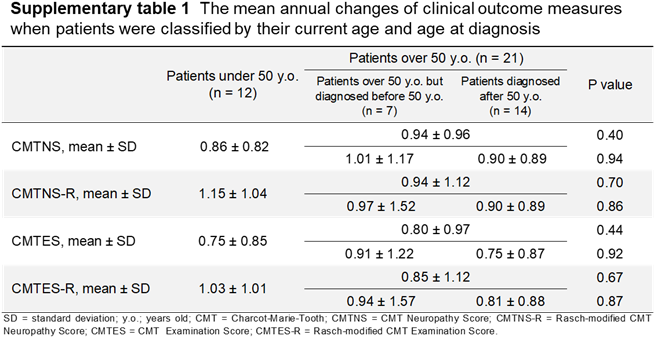

Supplement: Supplementary file 1 [file Image_1.TIF]
